# Correction to: Urinary extracellular vesicles prevent di-(2-ethylhexyl) phthalate-induced hypospadias by facilitating epithelial–mesenchymal transition via PFN2 delivery

**DOI:** 10.1007/s10565-024-09898-x

**Published:** 2024-08-12

**Authors:** Shibo Zhu, Xiangliang Tang, Jin Zhang, Jinhua Hu, Xiaofeng Gao, Dian Li, Wei Jia

**Affiliations:** Department of Pediatric Urology, Guangzhou Women and Children’s Medical Center, Guangzhou Medical University, Guangzhou, 510623 People’s Republic of China

**Correction to: Cell Biol Toxicol (2023) 39:2569–2586**

10.1007/s10565-023-09838-1

The original version of this article unfortunately contained an error.

In Figures 1, 2, 3, 5, 6, 7, and 8, the word "N-cadhrtin" was misspelled. It has been corrected to “N-cadherin”.

